# On the Use of the Value Added Tax for Redistributive Purposes in Italy

**DOI:** 10.1007/s40797-023-00224-8

**Published:** 2023-04-27

**Authors:** Federica Lanterna, Paolo Liberati

**Affiliations:** Department of Economics, University Roma Tre, Rome, Italy

**Keywords:** VAT, Redistribution, Cash transfers, Italy, H250, H200, H230

## Abstract

This paper shows that a Value Added Tax (VAT) with multiple tax rates in Italy is not the most effective way to pursue redistributive aims. As the tax revenue of the Italian VAT is significantly affected by the use of reduced tax rates and exemptions, we suggest that a shift to a uniform VAT coupled with cash transfers to households with children might better achieve redistributive targets. This outcome is also robust to alternative ways of targeting cash transfers to the poorest households. We also show that a uniform VAT with cash transfers, from a distributive point of view, is preferred to the extensive use of the zero rate on specific goods and services. Further improvement in the distributional results could even be achieved by considering that a uniform VAT may significantly reduce VAT evasion due to the difference of tax rates applied to the intermediate stages.

## Introduction

Value Added Tax (VAT) is one of the most important taxes currently in place in the European countries. It has received increasing attention since its recommendation by the Fiscal and Financial Committee of the European Economic Community in 1962 as a way to harmonise the sales tax system then prevailing among the member states. To some extent, this attention was stimulated by the official adoption of VAT by the French government in 1954, even though its use in Japanese local governments (prefectures) had previously been proposed by the Shoup Mission of 1949 (Kaizuka 1992). In particular, in Western Europe the adoption of VAT was suggested by the need to avoid the trade distortions associated with the widespread use of cascading indirect taxes (Ebrill et al. 2001). More recently, the Mirrlees Review also addressed the issue of how to properly use VAT in the context of an effective tax system (Mirrlees et al. 2011).

In Italy, VAT has been in place since 1972, when it was introduced as part of a more comprehensive reform of the overall tax system that took place in the 1970s. Although the original proposal was to use a national VAT with a uniform rate operating at the intermediate stage while leaving the final stage to local governments through the use of autonomous sales taxes, the final outcome was to use a central VAT with multiple tax rates on all stages, including sales to the final consumers.^1^ Currently, in Italy, the structure of VAT involves the application of four tax rates, 4, 5, 10 and 22%, giving rise to a tax revenue equal to slightly more than 6% of GDP, one of the lowest ratios among the European member states, notwithstanding the fact that the ordinary tax rate (22%) is one of the highest. However, since the 5% rate is applied to a very narrow set of goods, for instance sage and rosemary sold in mixed packs, some health provisions linked to the pandemic period, some education provisions by cooperatives, and a few other specific goods, in what follows we disregard the 5% tax rate.

Reforms of the value added tax in Italy have been discussed for many years, and the debate has focused mainly on whether to change the levels of the tax rates or their number, either for the purposes of redistribution or efficiency. Nevertheless, in the most recent past, revisions have been confined to changing the ordinary tax rate from 20 to 21% in 2011 and to 22% in 2013, mainly to deal with the Italian public finance constraints in the absence of alternative sources of funding the budget (Arachi et al. 2012). However, interest in modifying the structure of VAT persists, for at least two reasons. The first is represented by recent proposals to revise the overall Italian tax system, including the possibility of changing the number and the level of VAT rates, or the distribution of the tax base among tax rates, something that is referred to as a strategy of rationalisation of the VAT structure. The second, as stated within the EU 2020 strategy, arises from the European Commission recommendation to shift the tax burden from direct to indirect taxes, through an increase of VAT rates followed by a reduction of either personal income tax or social security contributions (for the case of Italy, see Curci and Savegnago 2019; Cammeraat and Crivelli 2020; Banca d’Italia 2021; for OECD countries, see Johansson et al. 2008). Further opportunities for a shift of the tax burden, including those involving a greater use of property and environmental taxes, are suggested by a number of studies, following the work of Johansson et al. (2008), which focuses on the potential positive effects on economic growth (European Commission 2011; Prammer 2011; Arnold et al. 2011; Alworth and Arachi 2012; Baiardi et al. 2018; IMF 2019).

In what follows, and with particular reference to the Italian experience, this paper will specifically address the distributive impact of VAT reforms and will provide empirical evidence that the use of reduced VAT rates is largely a weak and wasteful way to achieve redistributive aims. We will show that a preferable distributive outcome—compared to the current VAT structure—could be obtained by using a uniform VAT rate coupled with a targeted system of monetary transfers. It is also shown that such a system is superior to the application of the zero rate on distributional sensitive goods, thus confirming the findings of the British experience.

The structure of the paper is as follows. Section 2 gives an account of the most prominent literature on this topic and provides an overview of the main theoretical issues. Section 3 deals extensively with the data and the methodology used to perform redistributive analyses. Section 4 will show the redistributive outcome of both the current system and the hypothetical reforms. Section 5 concludes.

## Literature Review and Theoretical Issues

Any change of VAT—in Italy as well as in other countries—is often criticised for its possible adverse redistributive impact on the poorest households, despite the fact that economic theory suggests that a differentiation of tax rates should be implemented only in the absence of alternative ways of achieving those redistributive aims [namely, optimal income taxes and transfers, as in Atkinson and Stiglitz (1976, 1980), Deaton and Stern (1986), Besley and Jewitt (1995)].^2^ Furthermore, when optimal conditions are not verified, the practice of differentiating tax rates does not always lead to satisfactory results. The British experience, for example, suggests that cash transfers might be more effective than zero-rating in achieving redistributive outcomes, also because zero and reduced VAT rates might help people with particular tastes rather than being targeted at those with low overall resources (Mirrlees et al. 2011).^3^

In other countries where differentiation is applied, it is also observed that the richest part of the population—with few exceptions—consumes more of almost all goods, which means that the share of the implicit subsidy of reduced rates usually increases with income.^4^ This argument can also be found in studies on optimal taxation, where, in the case of normal goods, the poorest individuals may benefit more from an increase of monetary transfers than from reduced tax rates (Atkinson and Stiglitz 1980; Heady 1993), as has also been shown by a number of empirical studies (O’Donoghue et al. 2004; Decoster et al. 2010; Crawford et al. 2010; Leahy et al. 2011; Joumard et al. 2012; CPB 2013; OECD and Korea Institute of Public Finance 2014; Kaplanoglou 2015; Cirillo et al. 2021).

Other empirical studies, regarding the case of Italy, have shown that redistributive aims are not appropriately pursued by multiple tax rates, as a better impact could be obtained by either reassigning goods among tax rates (Gastaldi et al. 2017) or even reducing the number of VAT rates (Liberati 2001; Gastaldi et al. 2017; Cammeraat and Crivelli 2020), in both cases ensuring revenue neutrality.^5^ In the same vein, in a recent document published by the European Commission (2021), the recommendation was to limit the use of multiple tax rates and exemptions that do not achieve the desired redistributive aims; however, at the same time, the recent approval of Directive 2022/542 provides for the possibility of changing the use of both reduced and super-reduced tax rates—including the zero rate—to adapt their use to digital, health, and environmental aims.

This latter aspect is particularly important if one considers that, in Italy, the *policy gap*—i.e. the tax revenue that is lost due to the deviations from the standard regime (Keen 2013)—is above the average of the Euro area. This loss of revenue is explained by the fact that the tax base subject to the reduced tax rates (about 43%) is almost twice as much as that observable in other European countries (Borselli et al. 2012; European Commission, 2021). Furthermore, it is worth noting that the pure scheme of VAT at a uniform rate would be equivalent to a tax on the value of final consumption at the final stage. Thus, deviations from this scheme determine a gap from the standard regime, which depends on the monetary impact of multiple tax rates, exemptions, and differentiated tax treatments along the intermediate stages.^6^ As reported in estimates provided by the European Commission (2022), updated as late as 2020, in Italy the policy gap amounts to 55.7% of the potential VAT revenue, the highest level in the European Union after Spain (56.3%) and Greece (60.3%). It is also quite important to observe that the share of the policy gap due to the *tax rate gap*—i.e. to reduced tax rates—is the fifth highest in Europe after Austria, Malta, Cyprus and Ireland. Moreover, by adding the level of tax evasion—according to the C-efficiency index—Italy is the worst-performing country in Europe. This can be attributed to a problem of VAT design more than to a problem of VAT implementation, as a significant increase in C-efficiency and revenue could be obtained without increasing the VAT standard rate.^7^ This implies that the emphasis often placed on various types of fraud and evasion (like the issue of ‘carousel frauds’) may to some extent be overstated, and that the hesitancy to move towards a less differentiated VAT is essentially misplaced. Indeed, the remarkable size of the policy gap, in Italy as well as in other European countries, is one of the reasons why it is often recommended to apply VAT on a broad base either at a single rate or narrowing the applicability of reduced rates (European Commission 2011).

In assessing the impact of multiple tax rates, however, it is worth considering that the differentiation may also be motivated by either efficiency or sectoral reasons. From the perspective of efficiency, the differentiation of tax rates should follow the size of the elasticity of goods with respect to prices; on the other hand, a differentiation of tax rates could be based on a higher taxation of those goods that are complementary to leisure in order to compensate for the negative impact of direct taxes on the labour supply.^8^ As argued by de Mooji and Keen (2012), however, existing empirical knowledge gives little reason for a confident differentiation on these grounds; for example, it would have the paradoxical outcome of requiring food, since this is complementary to leisure, to be taxed more (Crawford et al. 2010).

From a sectoral perspective, the differentiation of tax rates is instead justified by the need to protect industrial and commercial activities that are particularly exposed to international competition; in this case, a lower tax rate is usually applied: (i) to take into account potential difficulties in shifting the tax burden on prices; (ii) due to the need to reduce the VAT burden on specific merit goods (e.g. health, education, and cultural activities); or (iii) the need to fight tax evasion in sectors with high labour intensity. Some of these reasons could lead to a permanent differentiation being kept, while others could either justify it only temporarily or not justify it at all.^9^

Whatever the prevailing reason, the European practice of applying VAT is strongly in favour of multiple tax rates, even though their application across countries is heterogeneous. Specifically, according to the list of VAT rates applied in the Member States of the European Union in May 2022, only Denmark applies a uniform VAT rate at 25%, while Bulgaria, Germany, Estonia, the Netherlands, and the Slovak Republic apply two rates.^10^ All other countries apply more than two rates, with Ireland and France using four tax rates.^11^

## Data and Methodology

### The Microsimulation Model

In order to investigate the distributive power of the current VAT structure, we use a static microsimulation model built on data from the Household Budget Survey (HBS) provided by the Italian National Institute of Statistics (Istat) for 2019.^12^ Since our analysis is essentially based on the analysis of VAT—and does not interact with other tax bases—we do not make use of other datasets such as the Survey of Households Income and Wealth (SHIW) and the Statistic on Income and Living Conditions (SILC).

HBS captures the spending behaviour of resident households, recording all expenditures made to purchase goods and services. In addition to expenditure items, the survey also includes economic, social and demographic characteristics of households. It contains data on expenditures at household level for 501 goods for a sample of 18,718 households corresponding to almost 26 million households in the whole country. The detail of the available data allows us to assign the corresponding VAT rate to each good, which makes it possible to analyse even extremely disaggregated hypotheses of tax reforms. In the few cases where a good surveyed in the HBS contains more than one good at VAT level with different tax rates, the good in the HBS has been assigned a weighted average tax rate, where the weights are given by the share of the elementary goods at VAT level.

Microsimulation models are among the most widely used tools for assessing the tax burden and redistributive effects of VAT, both in its statutory configuration and, in particular, in the case of reform scenarios. With regard to Italy, Baldini (2001) uses the MAPP98 model to study, among other institutional features of the Italian tax-benefit system, the impact of VAT, using data on 1998 incomes from SHIW; Arachi et al. (2012) analyse the impact of the fiscal consolidation policy adopted in 2011, using a static microsimulation model based on a specific matching between 2010 SHIW data and Istat household consumption data. Again, by integrating consumption and SHIW data, the Bank of Italy’s microsimulation model, BIMic, is extended to incorporate the role of VAT (Curci et al. 2017). More recently the Department of Finance has built the Value Added Tax Simulation Framework (VATSIM-DF), a model composed of two other models: VATSIM-DF (I), dedicated to the valuation of VAT revenues, and VATSIM-DF (II), whose aim is to estimate the distributional effects of VAT (see Calà et al. 2020 for the first module; Cirillo et al. 2021 for the second). In other countries, Kaplanoglou (2015) uses a microsimulation model to assess the redistributive impact of indirect taxes between 1988 and 2011 for Greece; Leahy et al. (2011) analyse the redistributive impact of VAT for Ireland, considering different scenarios. More generally, cross-country analysis is carried out using the EUROMOD model, which makes it possible to consider 27 European countries (see, among other studies, O’Donoghue et al*.* 2004; Decoster et al. 2010).

### Specific Assumptions

The use of microsimulation models involves a number of assumptions. First, reliable and timely information on household income is not available in HBS data. Thus, when necessary, the income distribution corresponding to the expenditure distribution surveyed by the HBS has been simulated using data on the average propensity to consume by equivalent income classes reported by Ilardi and Zanichelli (2020), and obtained by the ratio of average total consumption (including both durable and non-durable consumption) to average household income in each income class.^13^ Second, tax evasion is not taken into account in the model, as details are not available regarding how it may be distributed along the expenditure distribution.^14^ Third, in our model households do not change their consumption as a result of the simulated reform, i.e. they continue to purchase the same basket of goods and services. Fourth, and partly as a consequence of the previous assumption, we assume full pass-through of VAT changes to consumer prices.

This latter issue has been widely debated in the literature both theoretically and empirically. From a theoretical point of view, and in regard to different market settings, the main results are based—among many studies—on the work of Katz and Rosen (1985), Stern (1987), Besley (1989), Delipalla and Keen (1992), Baker and Brechling (1992), Cremer and Thisse (1994), and Fullerton and Metcalf (2002). From these studies it emerges that the analysis of pass-through may depend on different factors, such as the structure and size of the market, whether the change in VAT is general or limited to certain products, the nature of the commodities involved, and finally, the speed of price adjustments. Carbonnier (2005) also emphasises a possible asymmetric effect depending on whether VAT rates increase or decrease. The main tools employed for the empirical analysis are based on natural experiments using econometric techniques such as difference-in-differences (Carbonnier 2005 for France) or weighted fixed-effects estimation (Chirakijja et al. 2009 for the UK). In general, the empirical results confirm a partial or full pass-through to consumer prices. However, the heterogeneity of the results does not lead to a uniform conclusion, which is why the absence of clear and well-founded empirical evidence on alternative hypotheses to full pass-through leads many empirical works to rely on this hypothesis (CPB 2010).^15^

### Validation of the Model

Households’ final consumption only attracts part of the total VAT revenue: around 63 billion euros rather than around 134 billion euros in 2019. This difference is mainly due to the technical functioning of the VAT in the intermediate stages and to the number of tax credits that accrue to some categories of VAT operators (for past investigation on this issue, see Convenevole 2006). Only a small part of this difference, on the other hand, is due to a specific underestimation of the final consumption in HBS data. There are indeed VAT transactions that contribute to the total tax revenue but are not recorded in the final consumption of households. As reported by Corte dei conti (2021), the most relevant items are: (i) VAT paid by the public administration as a final consumer; (ii) the fraction of VAT paid by public administrations under the regime of split payment, by which the purchasers replace the sellers in paying VAT to the State; and (iii) the amount of VAT paid by VAT taxpayers under regimes of tax exemption, who either cannot deduct VAT on purchases or can deduct it only partially (*pro rata*), but are not final consumers. Moreover, official data from the tax administration are reported gross of VAT credits, while the imputation of VAT in our model implicitly excludes this item. Since we are interested in the redistributive impact of multiple VAT rates on households, we concentrate on the VAT that falls on final consumption.^16^

### Indices of Redistribution

In order to assess the redistributive effects of VAT, we will make use of tools that are widely employed in the literature. A first set of indicators will serve the aim of measuring the correlation between what the proper distribution of VAT rates among goods should be and the actual distribution of VAT rates. A second set of indicators, meanwhile, will measure the redistributive power of the current VAT structure and of the various hypotheses for reforms.

In the first case, two indicators will be used, distributional characteristics and Gini elasticities. Distributional characteristics ($DC$) represent a measure of how the consumption of any given good is concentrated along the income distribution, and can be defined as follows:

$${DC}_{i}=\frac{\sum_{h=1}^{H}{\beta }^{h}{x}_{i}^{h}}{{\overline{\beta }X}_{i}}$$

where ${x}_{i}^{h}$ is the consumption of good $i$ by individual $h$, ${X}_{i}$ is the total consumption of good $i$, ${\beta }^{h}=\frac{1}{{\left({x}^{h}\right)}^{e}}$ is a social weight attached to individual $h$ that depends on a parameter of inequality aversion $e$, $\overline{\beta }$ is the average social weight, and $H$ is the total number of individuals.^17^ The higher the value of $e$, the higher the degree of inequality aversion. Furthermore, for any given value of $e$, the higher the value of DC, the more concentrated the consumption of the good among the poorest. In general, when $e>0$, it is assumed that an increase of income allowed to poorer individuals has a higher marginal social value than the same increase allowed to richer individuals. For example, $e=1$ implies that a marginal unit of an increase of income for an individual who has twice as much income as another individual is worth half as much in terms of social value. The social value of an increase in income for the richest people is lower when $e$ increases, until it approaches a Rawlsian-type social welfare function [see, for example, the discussion in Madden (1995), and the general treatment by Stern (1977)].

With regard to the second indicator, Gini elasticity ($GE$), we follow Yitzhaki and Schechtman (2013) in defining $GE$ for each good *j*, as follows:

$${GE}_{j}={s}_{j}({Z}_{j}-1)$$

where ${s}_{j}=\frac{{\mu }_{j}}{{\mu }_{y}}$, the ratio between the average expenditure on good *j* and average income *y*; and ${Z}_{j}=\frac{Cov\left[{x}_{j},F(y)\right]}{Cov\left[y,F(y)\right]}\frac{{\mu }_{y}}{{\mu }_{j}}$, where ${x}_{j}$ is consumption of good *j* and $F(y)$ is the cumulative distribution function of the reference variable *y*. This term determines the sign of the Gini elasticity and its interpretation is made easier when expressed by the ratio between the concentration coefficient of commodity *j* and the Gini index of the ordering variable *y*, as follows:

$${Z}_{j}=\frac{{C}_{j}^{y}}{{G}_{y}}$$

A value of ${Z}_{j}>1$ implies that the concentration coefficient of expenditures on commodity *j* is greater than the Gini index of the ordering variable *y*. Thus, an increase of the tax rate on *j* will have a progressive impact, while a reduction of the same tax rate would be regressive. The opposite holds true when ${Z}_{j}<1$. In the special case where ${Z}_{j}=1$, the tax change will be neutral from a distributional perspective. Finally, ${Z}_{j}<0$ identifies an inferior good, leading the concentration curve to lie above the equidistribution line.

As regards the second set of indicators used to assess the redistributive effects of VAT, we make use of the Gini index, the Reynolds–Smolensky (*RS*) index and the Suits index. The Gini index (Gini 1912, 1914; Pietra 1915) takes values between zero (perfect equidistribution) and one (maximum inequality). Concentration indices, meanwhile, can take values between − 1, when the variable under consideration is concentrated in the hands of the poorest individual, and 1, when it is concentrated in the richest unit. Using the Gini index, the redistributive impact of any tax structure can be measured by the Reynolds–Smolensky index (Reynolds and Smolensky 1977), i.e. by the difference between the pre-tax Gini index $({G}_{Y})$ and the post-tax concentration index ${(C}_{Y-T})$:

$$RS= {G}_{Y}-{C}_{Y-T}$$

Note that in this definition, $RS$ is equal to the total redistributive effect (${RE=G}_{Y}-{G}_{Y-T}$) in the absence of re-ranking (*R*), which measures the re-balancing (i.e. the change of individual positions) that might occur in the shift from gross to net income distribution among taxpayers, and which is defined as $R=\left({G}_{Y-T}-{C}_{Y-T}\right)>0$. In the absence of re-ranking, ${G}_{Y-T}={C}_{Y-T}$, which means that $RS=RE$. When there is re-ranking, $RE=\left[{G}_{Y}-{C}_{Y-T}\right]-\left[{G}_{Y-T}-{C}_{Y-T}\right]=RS-R$.

In what follows, we will use a decomposition of the redistributive effect into three elements: (i) the Kakwani index (*K*); (ii) the average tax rate $\left(\frac{t}{1-t}\right)$; and (iii) the re-ranking effect. The Kakwani index (Kakwani 1977) measures the departure from proportionality of a given tax structure, and is obtained as the difference between the concentration index of taxes and the pre-tax Gini index $({{C}_{Y,T}-G}_{Y})$. When the tax is proportional, the Kakwani index is zero. Thus:

$$RE=K \left(\frac{t}{1-t}\right)-R={(C}_{Y,T}- {G}_{Y}) \left(\frac{t}{1-t}\right)-R$$

The higher $RS$ is, the greater the redistributive capacity of the system will be. On the other hand, the greater $R$ is, the lower the ‘pure’ redistributive effect of the system ($RE$) will be.

Finally, the Suits index (Suits 1977) is a measure based on the relation between the cumulative share of pre-tax income and the cumulative share of the tax burden. In this case, a proportional tax would generate a Lorenz curve overlapping the equidistribution line; while a progressive tax would generate a Lorenz curve below the equidistribution line. In the limiting case of a regressive tax, the Lorenz curve would lie above the equidistribution line. According to this framework, the Suits index is given by 1 minus the ratio between the value of the area below the Lorenz curve (*L*) and the value of the area below the equidistribution line (*Q*):

$${P}^{s}=1-\frac{L}{Q}$$

The index would be zero under a proportional tax; it would be positive with a progressive tax; and it would be negative with a regressive tax.

## Empirical Analysis

### The Correlation of VAT Rates with Distributional Indicators

**Fig. 1:**
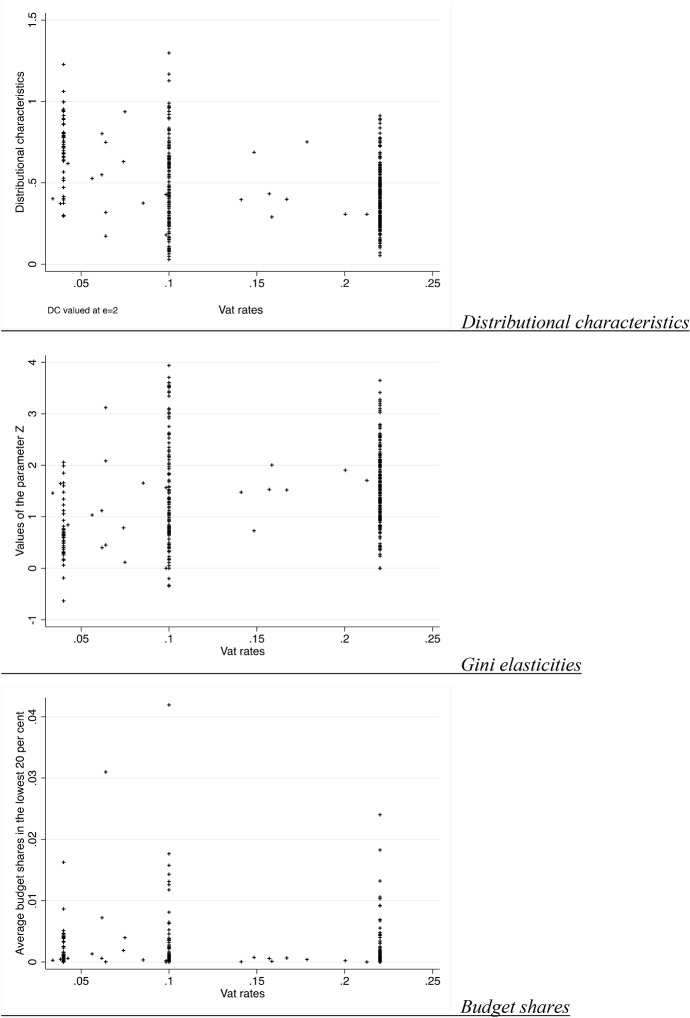
Distributional indicators and tax rates. Note: The top panel shows the relationship between average tax rates and distributional characteristics. The middle panel shows the relationship between average tax rates and the parameter of the Gini elasticity. The bottom panel shows the relationship between average tax rates and average budget shares. The points that are out of the statutory tax rates identify the average tax rates applied to groups of commodities including elementary items taxed at different rates. Source: Author’s elaborations on Istat data, 2019

In this section, the distributive rationality of the assignment of the current VAT rates is assessed against the distributional characteristics of the goods and the Gini elasticities, with the aim of verifying whether there is any correlation between them. As explained above, the meaning of distributional characteristics implies that goods with a higher value of DC should be assigned a lower VAT rate. Thus, if VAT rates were optimally targeted, we should observe the standard VAT rate (22%) applied to goods with lower values of DCs, and lower VAT rates (4 and 10%) to goods with higher values of DCs. Since the aim is to investigate distributional issues, we choose to analyse the relationship between VAT rates and the values of DC when $e=2$, which corresponds to considering a sufficiently high degree of inequality aversion, in order to find results that convey a social preference for redistribution. As shown in the top panel of Fig. 1, only a weak negative relationship is revealed by the data, which is also confirmed by a negative correlation of − 0.32.^18^ This outcome implies that, in principle, the redistributive outcome of multiple VAT rates could be improved if tax rates were reassigned to goods according to the values of DCs.^19^ A similar outcome is obtained when considering Gini elasticity. In this case, if VAT rates were properly targeted, high values of ${Z}_{j}$ should be associated with high VAT rates; but in this case too, a positive correlation appears only weakly in the middle panel of Fig. 1. Given that the two main distributional indicators show no significant correlation with current VAT rates, it is of some interest to investigate whether VAT rates have been set only on the basis of budget shares. In this case, one has to look at whether lower tax rates are applied to those goods with greater weight in the budget of the poorest households, represented by ${s}_{i}^{h}=\frac{{x}_{i}^{h}}{{x}^{h}}$, where ${s}_{i}^{h}$ is the share of good $i$ in total consumption of individual $h$. Since budget shares are specific to each household $\left(\frac{{x}_{i}^{h}}{{x}^{h}}\right)$, while distributional characteristics are based on the way in which expenditures are distributed and weighted among households—i.e. a sequence of $\left(\frac{{x}_{i}^{h}}{{X}_{i}}\right)$—the best strategy is to calculate average values for each good among the poorest part of the population. With this purpose in mind, we define poor households as being those below the 2nd decile of the expenditures distribution and—for each good—we calculate the average budget share of that fraction of the population.^20^ From a policy perspective, higher budget shares among the poorest should be associated with lower VAT rates. The bottom panel of Fig. 1 suggests that in this case too there is no clear negative pattern, as confirmed by a weak negative correlation coefficient (− 0.124).

It is worth observing at this stage that among the 20 goods with the highest DC at $e=2$, only two goods appear among those with a high budget share among the poorest. This implies that VAT reforms based on budget shares may conflict with reforms based on either *DC* or ${Z}_{j}$; the fact that a given good has a higher weight in the budget of the poor does not imply that it is consumed mostly by the poor (Navajas and Porto 1994). On the contrary, if consumption is proportionally higher for richer households, the implicit subsidy of applying the reduced VAT rate may not have the intended redistributive effects. It is therefore important, from a policy point of view, to clarify what the underlying target is when assessing the distributive impact of VAT rates and that of their possible reforms. In any case, our analysis shows that no indicator is strongly correlated with VAT rates, implying that multiple tax rates, in Italy, may be motivated by reasons that are at least partially different from that of achieving redistributive aims.

### The Redistributive Power of the Current VAT Structure

Following the findings of the previous analysis, a natural question to ask is whether a different targeting of VAT rates may provide a better redistributive outcome. Alternatively, one could investigate whether dropping any redistributive purpose using VAT can be compensated for by other more effective tools, as monetary transfers to the poorest households. Before going any further, and for a proper comparison with alternative VAT structures, it is worth analysing the redistributive power of the current VAT using more standard indicators. The first is the path of the average tax rate across quantiles, which requires us to address the long-standing issue of whether to use total expenditures or total income as the denominator. In order to give a complete picture of the impact of VAT, the average tax rate is estimated by considering percentiles of both total net expenditures and a proxy of the disposable income, estimated—as mentioned before—on the basis of the average propensity to consume provided by Ilardi and Zanichelli (2020).^21^

**Fig. 2:**
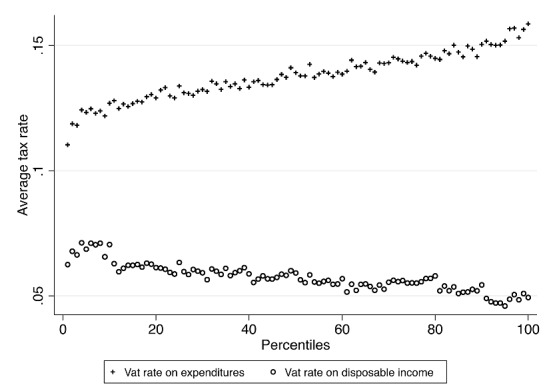
The pattern of average VAT rates in the current system. Note: The graph depicts the pattern of the VAT average tax rate over percentiles of the expenditure distribution and the income distribution. In the first case, the average tax rate is measured by the ratio between VAT and total expenditures net of VAT; in the second case, the average tax rate is measured by the ratio between VAT and disposable income. Source: Author’s elaborations on Istat data, 2019

Figure 2 reports the expected outcome, showing VAT progressivity when measured over expenditures, and VAT regressivity when measured over disposable income. It is worth noting that while for the first decile the gap between expenditures and income is narrow—as expenditures here are close to total income—for the other deciles the difference is increasing. In terms of levels, in the current system the average tax rate in the first decile is about 12.7%, while in the last decile it increases to 15.8% when measured over expenditures (Table 1, panel A, column (a)). Things change drastically when average tax rates are measured over income, ranging from about 10.2% in the first decile to 4.9% in the last one (Table 1, panel B, column (a)). The results on VAT incidence, when measured over disposable income, are in line with the findings of Cirillo et al. (2021). The authors find that the VAT incidence for the lowest income decile is equal to 4.8%. However, they report a higher incidence for the highest income decile (13.8%), thus suggesting a greater regressivity of VAT.^22^

**Table 1:** Average VAT rates of the current system and of simulated reforms

Deciles of equivalent expenditures	Current system	One-rate VAT (no cash transfers)	One-rate VAT (with cash transfers)	Zero rate on some goods
	(a)	(b)	(c)	(d)
*(a) On expenditures*				
1	0.127	0.150	−0.112	0.123
2	0.133	0.150	0.112	0.131
3	0.136	0.150	0.160	0.134
4	0.139	0.150	0.160	0.137
5	0.142	0.150	0.160	0.140
6	0.144	0.150	0.160	0.143
7	0.146	0.150	0.160	0.146
8	0.149	0.150	0.160	0.149
9	0.152	0.150	0.160	0.153
10	0.158	0.150	0.160	0.161
*(b) On disposable income*				
1	0.102	0.120	−0.090	0.099
2	0.066	0.075	0.056	0.065
3	0.063	0.070	0.074	0.062
4	0.063	0.068	0.072	0.062
5	0.060	0.064	0.068	0.060
6	0.059	0.061	0.065	0.059
7	0.055	0.057	0.061	0.055
8	0.058	0.059	0.062	0.058
9	0.054	0.054	0.057	0.055
10	0.049	0.047	0.050	0.050

Source: Author’s elaborations on Istat data, 2019

The second indicator is given by the standard Gini and concentration coefficients of the various VAT parameters measured on households ranked by equivalent expenditures.^23^ Data in the first panel of Table 2 (column (a)) report a concentration coefficient of VAT of 0.278 against a concentration coefficient of the tax base of VAT equal to 0.246, which means that VAT is more unequally distributed than total expenditures, thus contributing to a certain—albeit modest—progressive effect on total expenditures.^24^ Obviously, this redistributive impact disappears if the same coefficients are calculated on equivalent disposable income. But even the progressivity measured on total expenditures is the outcome of different patterns of the concentration coefficients of the three VAT rates; in particular, only the standard tax rate of 22% exhibits a concentration coefficient higher than that for total expenditures (0.312 vs. 0.246), while the concentration coefficients of 4 and 10% (0.143 and 0.208, respectively) suggest that VAT applied with those tax rates is more equi-distributed than total expenditures, meaning that the implicit subsidy of the reduced tax rates significantly benefits the richest part of the population as well. Support for this conclusion also comes from the lower levels of the standard Reynolds–Smolensky index (0.003), of the Kakwani index (0.037), and of the Suits index (0.041), as reported in the second panel of Table 2, column (a).

**Table 2:** Concentration coefficients and redistributive indices: current system and simulated reforms

	Current system	One-rate VAT (no cash transfers)	One-rate VAT (with cash transfers)	Zero rate on some goods
	(a)	(b)	(c)	(d)
*Concentration coefficients (ranking by equivalent expenditures net of VAT)*				
Total expenditures net of VAT	0.246	0.246	0.246	0.246
VAT	0.278	0.246	0.351	0.284
Super-reduced tax rate	0.143	–	–	0.132
Reduced tax rate	0.208	–	–	0.214
Standard tax rate	0.312	–	–	0.318
*Gini and redistributive indices*				
Gini (gross of VAT)	0.313	0.311	0.317	0.314
Gini (net of VAT)	0.311	0.311	0.311	0.311
RS index	0.003	0.000	0.006	0.003
Kakwani index	0.037	0.000	0.073	0.042
Suits index	0.041	0.000	0.059	0.045
Confidence intervals Gini gross	[0.3090 0.3182]	[0.3072 0.3146]	[0.3123 0.3207]	[0.3099 0.3180]
Confidence intervals Gini net	[0.3064 0.3154]	[0.3066 0.3152]	[0.3069 0.3149]	[0.3065 0.3153]
Confidence intervals RS (difference)	[0.0026 0.0028]	[-0.0006 0.0007]	[0.0054 0.0058]	[0.0026 0.0035]

Note: The confidence intervals are evaluated at the 95% level. We report, again, the RS index with the indications of more decimals to show the significance. Thus, in the current system the RS index is equal to 0.0027; in the One-rate VAT (no cash transfers) scenario it is equal to 0.000; in the third reform it is equal to 0.0058 and in the last scenario it is equal to 0.0031. In each case, except in the second reform, the RS index is significative. Source: Author’s elaborations on Istat data, 2019

### A One-Rate VAT

**Fig. 3:**
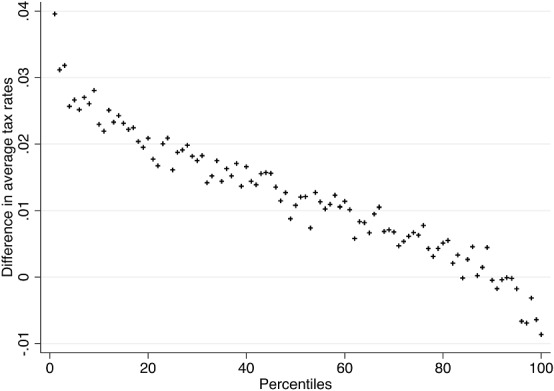
Difference of average tax rates between a uniform VAT at 15% and current VAT rates. Note: The graph plots the difference, for each percentile, between the average tax rate in the case of uniform VAT (15%) and the average tax rate of the current system. In both cases, average tax rates are measured by the ratio between VAT and total expenditures net of VAT. Source: Author’s elaborations on Istat data, 2019

The previous results suggest that the differentiation of VAT rates might not be particularly useful when pursuing redistributive aims. This conclusion is in fact only partially true, and this can be demonstrated by simulating the differential impact of a one-rate revenue-neutral VAT. According to our elaborations, an almost revenue-neutral one-rate VAT, replacing the current system, should be applied with a tax rate of 15%. The outcome of this simulation is reported in Fig. 3, measuring—for each percentile of net expenditures—the difference between the uniform 15% VAT rate and the actual three-rate structure. As can easily be observed, the greatest benefit from a one-rate VAT is strongly concentrated in the highest percentiles, where this difference is negative, implying—as expected—that the richest households may be less burdened by VAT when it is applied on a uniform basis.

This conclusion might be an obstacle to supporting VAT reforms aimed at abolishing the use of multiple tax rates, as a non-negligible adverse impact—between 2 and 3 percentage points of the average tax rate—is concentrated in the first two deciles. The issue then becomes that of assessing whether the undesirable redistributive impact can be compensated for by using different tools. To some extent, the application of a uniform VAT rate, leaving income tax and social benefit schemes to address distributional concerns, is increasingly recognised as a policy strategy as well (Cnossen 2022). Furthermore, it has also been shown that a uniform VAT rate offset by a reduction in the standard rate may foster economic growth more than an increase in the standard rate coupled with a reduction of income taxes (Acosta-Ormaechea and Morozumi 2019).

Two useful ways to compensate for the adverse distributive impact of a uniform VAT are either to strengthen the provision of public goods and services aimed at supporting the poorest households, or to increase the use of cash transfers. For our purposes, we will prove that, to some extent, monetary transfers targeting the neediest households can balance the adverse redistributive impact of uniform VAT. This strategy, however, needs to be funded; thus, our simulation will concentrate on the possibility of applying a uniform VAT rate at the same time that additional tax revenue is provided with which to pay monetary transfers. In particular, our aim is to have as much as the amount of the existing cash transfers (*Assegno per il Nucleo Familiare*, ANF) to households with children in 2019 (about 6 billion euros). The uniform tax rate that allows us to achieve this aim is 16%. In our simulation, cash transfers are paid to households with children by defining an amount that increases according to the implicit equivalence scale used by Istat in defining the poverty thresholds.^25^ In particular, the total amount of cash transfers $\theta$ that can be paid to households is given by $R= \theta \left[{n}_{1}\left({h}_{1}\right)+{n}_{2}\left({h}_{2}\right)+{n}_{3}\left({h}_{3}\right)+{n}_{4}\left({h}_{4}\right)\right]$, where *R* is the total amount of resources available (6 billion euros), ${n}_{i}$ is the coefficient of the implicit equivalence scale needed to re-evaluate the base amount—with ${n}_{1}=1$ and ${n}_{k}>1$ and increasing with the number of children $k$—and ${h}_{k}$ is the number of households with *k* children, which identifies the number of households to which $\theta {n}_{k}$ has to be paid. From the previous expression, the base amount will be given by $\theta =\frac{R}{{\sum }_{k}{n}_{k}{h}_{k}}$, which in our simulation amounts to 180 euros on a monthly basis for a household with one child, which is then increased by the equivalence scale, ending with 388 euros on a monthly basis for households with 4 or more children. In performing this analysis, we neglect the additional funds that a uniform VAT rate may require by limiting the VAT evasion that depends on the possible different tax rates applied to sales and purchases in B2B transactions, and by reducing the administrative costs due to reimbursements when the VAT rate on sales is lower than the VAT rate on purchases. These elements are strategically important for VAT reforms, as VAT evasion due to multiple tax rates in the intermediate stages is quantified at about 10 billion euros, i.e. about 1½ times the additional resources that we estimate by using a uniform VAT of 16%.^26^ In this regard, Nens (2021) also suggests that a uniform VAT, either at all stages or in the intermediate stages, may contribute to reducing the amount of VAT evasion.

Now, by considering only a uniform VAT, the picture presented in Fig. 3 would have an upward shift, causing an additional burden to each household, albeit with a decreasing intensity. When considering both tools—a uniform VAT and monetary transfers—the outcome is significantly different. In particular, we design the payment of monetary transfers according to the poverty thresholds by household size defined by Istat, as reported in Table 3, which also reports the amount of cash transfers assigned to households with children.^27^ In order to analyse the impact of this policy in more detail, we use a version of the sequential marginal dominance to capture the impact of the reforms decomposed by family size.^28^ This tool allows us to assess the impact of the distribution of a monetary transfer by considering its effects in two dimensions: household ability and household need. This two-dimensional ranking assumes that the least able and the neediest households are to be considered more deserving of a marginal income transfer. In our case, the level of household consumption expenditure is taken as a proxy of the level of income, and household size is taken as a proxy of an indicator of needs. In order to assess the impact of a policy involving a monetary transfer, a welfare measure is identified.

**Table 3:** Poverty thresholds and amount of cash transfers

Household size	Poverty thresholds (monthly amounts in euros)	Implicit equivalence scale	Cash transfers (monthly amounts in euros)
1	656.97	1.00	0
2	1094.95	1.67	180
3	1456.28	2.22	240
4	1784.77	2.72	293
5	2080.41	3.17	342
6	2365.09	3.60	388

Note: Relative poverty thresholds are defined by Istat following the poverty line of the International Standard of Poverty Line (ISPL), which determines as poor a family with two members with a consumption expenditure lower or equal to the per capita average consumption expenditures. To define the corresponding poverty lines of families with more than two members, Istat uses the equivalence scale by Carbonaro (1985). The implicit equivalence scale in the third column is derived from the definition of poverty thresholds by Istat, reported in the second column. Source: Authors’ elaborations, 2019

In particular, we define ${dB}^{h}=\left[\left(\sum_{i}{x}_{i}^{h}{t}^{U}-{c}^{h}\right)-\sum_{i}{x}_{i}^{h}{t}_{i}\right]$, where ${t}^{U}$ is the uniform tax rate and ${c}^{h}$ is the cash transfer aimed at compensating for the burden of the uniform VAT. The term in round brackets is the VAT paid by each household at the uniform VAT rate net of the cash transfers it receives; the second term, meanwhile, is the VAT paid under the current regime. Thus, for each household *h*, the previous expression measures either the gain (${dB}^{h}<0$) or the loss (${dB}^{h}>0$) of moving to a uniform tax rate and coupled with a cash transfer, compared with the existing three-rate structure. Accordingly, we can then define the cumulative change by $\sum_{h\in \overline{H}}{dB }^{h}$ for all households of a given type $\overline{H }$. For this purpose, households have been split according to the number of children—assumed as an indicator of need—and ranked according to their total consumption. Then, the cumulative sum has been sequentially calculated by progressively adding household types starting from those with the highest number of children: more than three children (3 + children); at least two children (2 + children); at least one child (1 + children); at least two members. Given the definition of ${dB}^{h}$ above, desirable reforms from a distributional perspective will require the condition $\sum_{h\in \overline{H}}{dB }^{h}\le 0$ for any household type. This condition will ensure that the cumulative burden of a tax-benefit system combining a uniform VAT and cash transfers will be lower than the burden caused by the existing three-rate VAT structure.

**Fig. 4:**
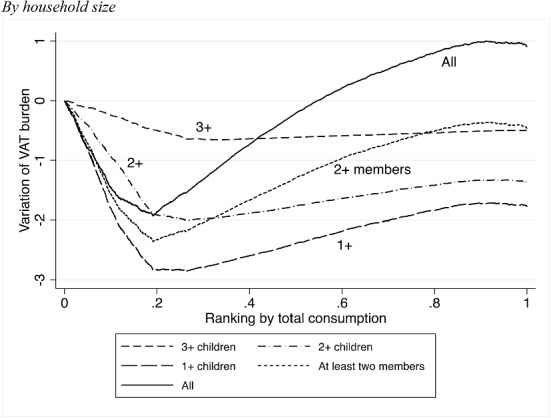
Cumulative gains and losses of uniform VAT plus cash transfers. Note: The decreasing shape means that when moving to the right of the ranking, households are cumulating gains from the reform; on the other hand, when the cumulative curve starts to increase, it means that households are added for whom the reform is producing losses. However, in cumulative terms, so long as the curve lies in the negative quadrant, cumulative gains are greater than cumulative losses. Source: Author’s elaborations on Istat data, 2019

According to this approach, we are able to identify whether the redistributive impact of the reform could either positively or adversely affect households of different sizes. Figure 4 gives the cumulative gain (if negative) or loss (if positive) obtained by the algebraic sum of two variables: the first is the net VAT paid by each household with a uniform tax rate and the cash transfer; the second is the VAT paid under the current three-rate structure. As can be seen, this cumulative difference is negative and decreasing in the lowest part of the ranking (the poorest). The decreasing shape means that when moving to the right of the ranking, households are cumulating gains from the reform, i.e. households with ${dB}^{h}\le 0$ are added; on the other hand, when the cumulative curve starts to increase, it means that households are added for whom the reform is producing losses, i.e. ${dB}^{h}\ge 0$. However, in cumulative terms, so long as the curve lies in the negative quadrant, cumulative gains are greater than cumulative losses; this occurs for each type of household with children, and it can be observed by an increasing negative cumulated sum when households are progressively added. Only when the overall population is considered does the cumulative difference end up in the positive quadrant. This implies that the reform cannot be positively assessed by all households in redistributive terms; yet, for our purposes, it means that for the poorest households a reform combining a uniform VAT rate and cash transfers to households with children may redistribute purchasing power better than the current VAT structure with multiple tax rates; and this poses serious questions about the power of pursuing redistributive aims with VAT.

It is worth noting that, in Italy, the possibility of limiting the use of the tax system for redistributive purposes while moving to public spending has also been recently introduced in a proposal for the revision of personal income tax, which would replace tax credits for dependent children with an increase of child benefits (Pellegrino 2021). Although in this case one should consider that tax credits for dependent children are not only a redistributive tool but also a way to define the appropriate ability to pay among taxpayers with and without children, these kinds of reforms highlight the need for the tax system to be as general as possible. Our findings, indeed, go in that direction, suggesting—more broadly—that the use of public spending could be more effective than the use of the tax system to achieve a satisfactory level of redistribution with revenue constraints.

The positive outcome of a uniform VAT combined with cash transfers can also be observed by looking at the more standard average tax rates in Table 1, and both the concentration coefficients and redistributive indices in Table 2. As regards average tax rates, Table 1 (column (c) in panel A) shows that the reform may increase progressivity over expenditures and even recover some progressivity over income (Table 1, panel B, column (c)) in the lowest part of the income distribution, surely better than what is achieved by the current system. In terms of average tax rates, it is worth noting that in both cases our reform would end up—on average—with paying a subsidy to households in the first decile. In terms of redistributive indices (Table 2), the impact of this reform is twice as much as the impact of the current system, with a Reynolds–Smolensky index of 0.006 compared to 0.003 of VAT with multiple tax rates. If nothing else, this simulation is able to show that the pursuit of redistributive aims should rely more on public expenditure tools, rather than on deviations from the standard VAT regime. Indeed, the undesirable redistributive effects of taxes may be more easily compensated for by using the other side of the public budget rather than fragmenting the tax system.

### Some Robustness Analysis

Support for the previous conclusions arises from a number of robustness analyses. The first considers the possibility of changing the structure of the cash transfer by using equivalent income instead of equivalent expenditures. By applying a cash transfer of 170 euros per month (close to the basic amount of the previous hypothesis), linearly decreasing with equivalent income until 1168 monthly euros of equivalent income (corresponding to the highest income in the third decile), one can again achieve a dominance of the cumulated benefits until the sixth decile of equivalent income, with more pronounced net benefits in the lowest part of income distribution.^29^

Second, in order to take into account the fact that our results are implicitly based on perfect targeting, we repeat the analysis considering the possibility of a partial take-up of monetary transfers. For this purpose, following the prevailing literature on *Reddito di Cittadinanza* (RdC) and *Assegno Unico e Universale* (AUU), we use different take-up rates to verify the robustness of the redistributive effects of cash transfers (Vittoria 2020; Baldini and Gallo 2021; Biagetti et al. 2022; Giuliano et al. 2022). Results are reported in Appendix (Table 10), where it is shown that only a very low take-up rate (61%) would be able to significantly weaken the redistributive impact of the uniform VAT rate with cash transfers.

Third, an attempt has been made to investigate whether the results are robust to certain aggregate behavioural reactions by households. For this purpose, the own-price elasticities for the groups of goods and services estimated in Regmi and Seale (2010) have been used to correct the VAT revenue after adjusting the tax base. By considering these elasticities, it is estimated that the VAT revenue may decline by about 255 million euros when moving to a uniform VAT; a reduction that may increase to 333 million euros in the hypothesis that the benefit is not shifted to consumers when the uniform VAT rate is lower than the actual VAT rate. In both cases, these adjustments would presumably involve less than 1% of the simulated VAT revenue. Overall, this loss of revenue does not significantly compromise the distributive impact of the reform, as the concentration coefficient is almost of the same magnitude as that calculated in the absence of behavioural reactions.

### Could a Zero Rate Improve the Redistributive Power of VAT?

The previous reform aims at compensating for the additional burden of a uniform VAT through the use of cash transfers. Now, a useful alternative to investigate is whether the same effect can be achieved when the same tax structure continues to be used. For this purpose, this section will address an alternative scenario where a zero rate is applied to specific goods and services.^30^ As a first step, we simulate the application of a zero rate to a basket of goods similar to that used in Britain, where this method is principally applied, and that includes pharmaceutical products, some food, services to children and the disabled, and some services in building and transport sectors.^31^ Trying to match this list with that available for the Italian case, we succeed in identifying 16 goods, reported in Table 4. It is worth noting that among these, and according to the Gini elasticity ${Z}_{j}$, 10 goods potentially deserve a reduced tax rate (${Z}_{j}$ < 1). Thus, the application of a zero rate could in principle yield a positive distributive outcome.

**Table 4:** Current treatment and Gini elasticities of the simulated zero-rated goods as in the British system

Goods and services	Current VAT rate (%)	Values of K
Equipment for disabled people	Exemption	> 1
Magazines	3.4	> 1
Books (not for school)	4.0	> 1
Children’s painting and picture books (it contains three distinct goods in Italian classification)	4.0	< 1 and > 0
Newspapers	4.0	> 1
Pharmaceutical products	10.0	< 1
Cesspools, septic tanks or similar (domestic)—emptying	10.0	< 1
Sewerage services supplied to domestic or industrial customers	10.0	< 1
Water supplied to households	10.0	< 1
Clothes for children	22.0	< 1
Babywear (this contains two distinct goods in the Italian classification)	22.0	< 1
Children’s clothes and footwear (this contains two distinct goods in the Italian classification)	22.0	< 1
Sanitary products	22.0	> 1
*Further zero-rated goods*		
Baby food	10.0	< 0
Fuel for heating (this contains two distinct goods in the Italian classification)	10.0	< 0

Note: The list of simulated zero-rated goods is derived from matching the data available in the Italian survey with those for the British system. Source: Author’s elaborations, 2019

**Fig. 5:**
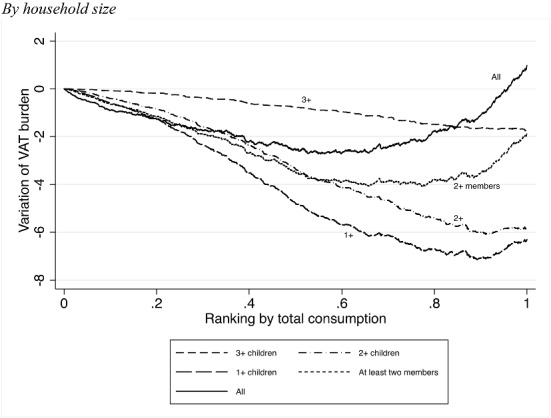
Cumulative gains and losses of VAT with zero-rated goods. Note: The decreasing shape means that when moving to the right of the ranking, households are cumulating gains from the reform; on the other hand, when the cumulative curve starts to increase, it means that households are added for whom the reform is producing losses. However, in cumulative terms, so long as the curve lies in the negative quadrant, cumulative gains are greater than cumulative losses. Source: Author’s elaborations on Istat data, 2019

**Fig. 6:**
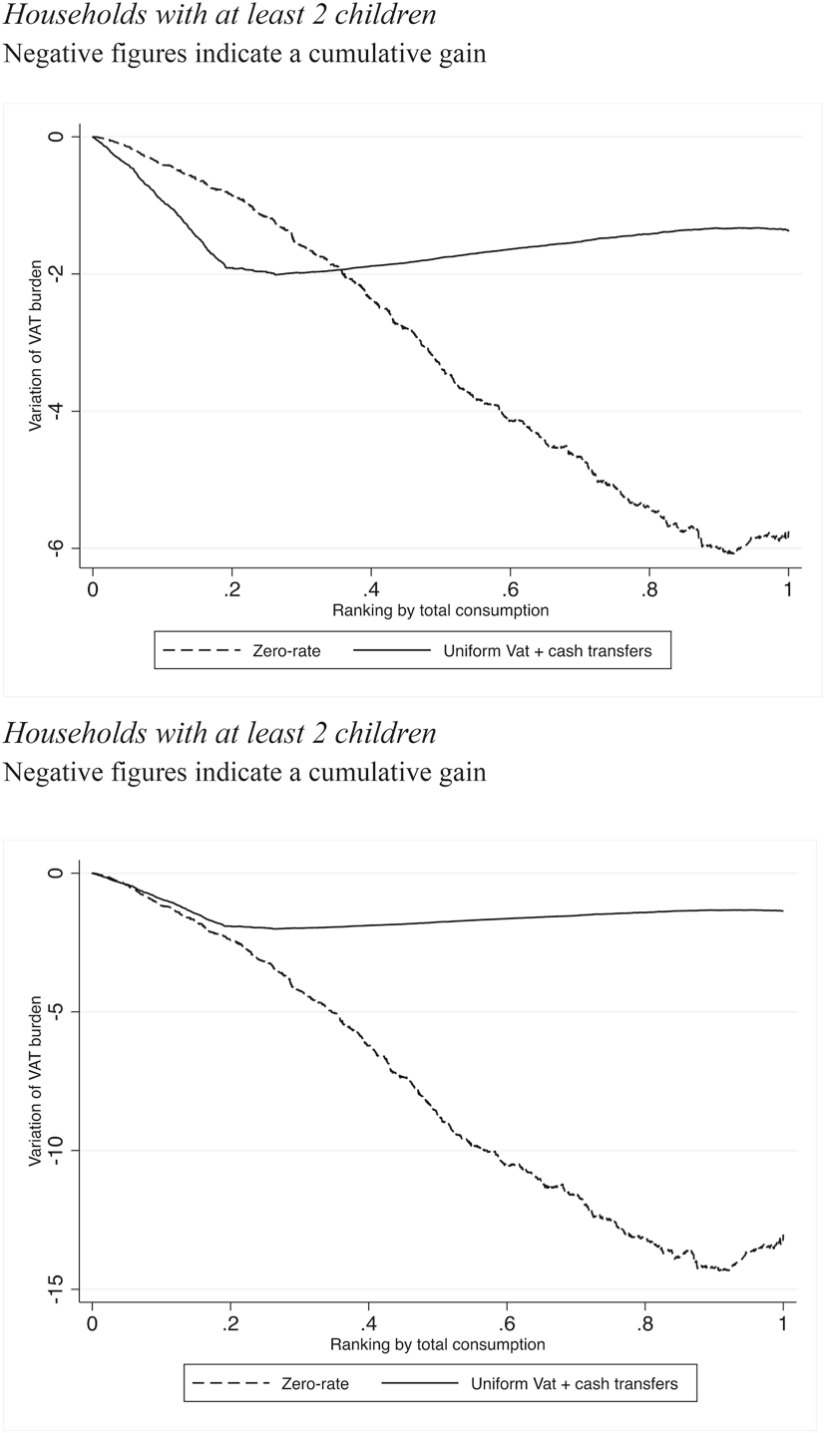
Comparison of cumulative gains and losses of VAT with zero-rated goods and uniform VAT plus cash transfers. Note: In the first graph the level of the VAT standard rate in the zero-rate reform is equal to 23.2%; in the second graph, meanwhile, it is equal to 23.4%. Source: Author’s elaborations on Istat data, 2019

Applying a zero rate to those goods, while maintaining the current three-rate structure, would cause a loss of revenue of about 2 billion euros, which we recover by increasing the standard rate to 23.2% to preserve revenue neutrality. Compared to the current system, the outcome of this reform is to slightly reduce the average tax rates in the first deciles and increase it in the highest two deciles (Table 1, panel A, column (d)), by making the system more “progressive” when measured over expenditures. However, when measured over income, Table 1—in column (d) of panel B—reveals that a zero rate does not help to shift the regressivity of VAT into progressivity. Support for this conclusion comes also from observation of the *RS* index, as the redistributive impact of this reform (Table 2, column (d)) is estimated to be low (0.003), even though the degree of progressivity—measured by the Kakwani index—improves slightly when compared with the existing structure (0.042). This improvement, however, is qualitatively different from that obtained in the previous reform proposal (uniform VAT and cash transfers). As reported in Fig. 5, using a zero rate might have positive redistributive implications; however, unlike what happens with the previous reform, the cumulative gains are much less concentrated in the poorest households, which means—once again—that the use of reduced tax rates is not as effective as using monetary transfers on the public expenditure side. This outcome is exemplified in the first graph of Fig. 6 for households with at least two children. As can be seen, the zero-rate policy gives a greater cumulative gain, as the curve is decreasing over the whole interval; however, by looking at the first four deciles, the cumulative gain is greater with a uniform VAT coupled with cash transfers. In other words, as is typical of multiple tax rates, the zero rate gives benefits also to the richest households with children, an impact that is avoided when using a selective cash transfer. Moreover, the greater cumulative gain of the zero rate is ‘bought’ at the cost of an ordinary tax rate of about 23.2%, while the impact of the uniform VAT plus cash transfer requires a milder 16%.^32^

In order to achieve the same effect in the lowest part of the income distribution, the zero-rate policy has to be extended to other goods. In particular, we simulate the possibility that a zero rate is additionally applied to fuel for heating and baby food, which are goods with a value of ${Z}_{j}$ < 0. The additional loss of tax revenue caused by adding these two goods is funded by further increasing the standard tax rate to 23.4%.^33^ In this case, again considering households with at least two children, the second graph in Fig. 6 shows that the impact on the lowest part of the income distribution is similar; again, the zero rate continues to cumulate relevant gains also in the upper part of the expenditure distribution, at the ‘price’ of a standard rate that is more than seven percentage points higher than in the case of uniform VAT. To some extent, this outcome only gives further support to what was argued by Titmuss (1958): namely, that the use of the tax system—compared with cash transfers—might cause a *hidden* welfare state by which implicit subsidies can be paid to richer households that would not be paid if they were made explicit as a money transfer.

**Fig. 7:**
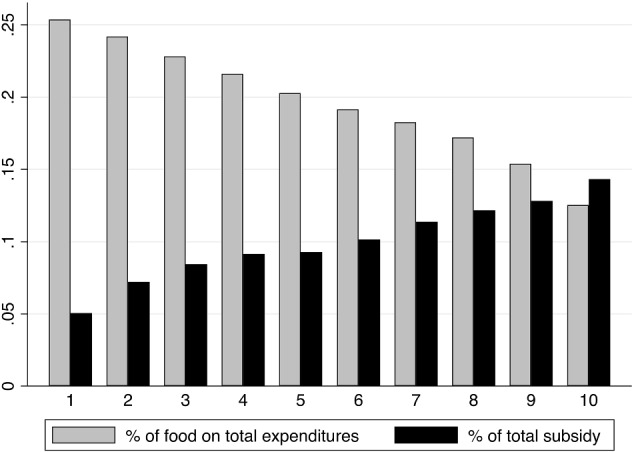
The implicit subsidy of the reduced tax rates on food. Note: The implicit subsidy—for a constant level of spending—is calculated by the difference between the standard rate and the reduced rate actually applied on food items. Source: Author’s elaborations on Istat data, 2019

Just to give empirical evidence for Italy of what may happen when applying reduced tax rates to some groups of commodities, we can use the current VAT structure to calculate the implicit subsidy of taxing food consumption at reduced rates (currently mostly at 4 and 10%, instead of 22%). The implicit subsidy—for a constant level of spending—is calculated by the difference between the standard rate and the reduced rate. Figure 7, by expenditure deciles, reports the percentage of food expenditures out of total expenditures (the grey bars), and the share of the implicit subsidy that each decile obtains by paying the current reduced tax rates rather than the standard VAT rate on food (the black bars). While—as expected—the weight of expenditures on food is declining across deciles, the share of the implicit subsidy is increasing, suggesting that the richest get a higher proportion of the total benefit arising from reduced tax rates. As it stands, it is rather surprising that this path of the implicit subsidy may be an argument invoked to defend the appropriateness of the distributive impact of reduced tax rates.

## Concluding Remarks

Even though VAT is often applied with multiple tax rates, its redistributive impact is low. In this paper, we have shown that, in Italy, replacing the existing three-rate structure with a uniform VAT, combined with an increase of cash transfers, may lead to better redistributive impacts. In particular, since the adverse effect of uniform VAT appears concentrated in households with children, we design cash transfers targeted to these on the basis of the poverty thresholds defined according to household size. In general terms, the positive redistributive result that we have obtained suggests that the use of the tax system to pursue redistributive aims may not be the best solution, and that better outcomes could be obtained by a more extensive use of public spending. This finding is also robust to alternative tax reforms, as in the case of the application of a zero rate to distribution-sensitive goods. Thus, what reasons there may be for multiple tax rates should be found in aims other than redistributive purposes.

## Data Availability

Raw data are generated at the National Institute of Statistics (Istat) and can be accessed only after authorization by Istat. The findings of this study are based on the author’s elaborations on those data by a microsimulation model.

## Appendix

See Tables 5, 6, 7, 8, 9 and 10.

**Table 5:** VAT rates in Europe

	Standard rate	Reduced rate	Super-reduced rate/parking rate
Austria	20	10/13	–/13
Belgium	21	6/12	–/12
Bulgaria	20	9	–
Croatia	25	5/13	–
Cyprus	19	5/9	–
Czech Republic	21	10/15	–
Denmark	25	–	–
Estonia	20	9	–
Finland	24	10/14	–
France	20	5.5/10	2.1/–
Germany	19	7	–
Greece	24	6/13	–
Hungary	27	5/18	–
Ireland	23	9/13.5	4.8/13.5
Italy	22	5/10	4/–
Latvia	21	5/12	–
Lithuania	21	5/9	–
Luxemburg	17	8	3/14
Malta	18	5/7	–
The Netherlands	21	9	–
Poland	23	5/8	–
Portugal	23	6/13	–/13
Romania	19	5/9	–
Slovakia	20	10	–
Slovenia	22	5/9.5	–
Spain	21	10	4/–
Sweden	25	6/12	–
UK	20	5	0
EU-average	21.46		

Note: Parking rates are applied by some EU countries to certain supplies of goods and services that are not included in Annex III of the VAT Directive. Source: European Commission, https://europa.eu/youreurope/business/taxation/VAT/VAT-rules-rates/index_it.htm#shortcut-8

**Table 6:** Main variables in the microsimulation model

*Number of households*	25,824,933
Total expenditures	825.4
Total expenditures net of VAT	761.3
Total tax base	433.2
*Tax base at 4%*	68.9
*Tax base at 10%*	162.5
*Tax base at 22%*	201.7
Total expenditures VAT exempt	392.3
VAT tax revenue	63.4
*Tax revenue at 4%*	2.8
*Tax revenue at 10%*	16.2
*Tax revenue at 22%*	44.4

Source: Author’s elaborations on Istat data, 2019

**Table 7:** Breakdown of expenditures by categories

Foods and non-alcoholic beverages	144.5
Alcoholic drinks and tobacco	14.3
Clothes and shoes	35.7
Home, water and gas	301.1
Furniture	35.5
Sanitary services	37.9
Transports	83.5
Communications	19.5
Entertainment and cultural activities	41.3
Education	7.1
Hotels and restaurant	36.8
Other goods and services	63.7
Total	820.9

Source: Author’s elaborations on Istat data, 2019

**Table 8:** Descriptive statistics

Deciles of equivalent expenditures	Number of households	Average VAT		Average total consumption
*(a) Current system*				
1	2,582,827	71.68		1051.20
2	2,583,828	105.59		1479.20
3	2,583,054	128.60		1767.27
4	2,582,461	149.37		2021.89
5	2,581,184	167.06		2265.53
6	2,581,761	195.56		2572.82
7	2,584,005	227.24		2944.04
8	2,581,265	258.93		3295.85
9	2,582,073	304.27		3851.50
10	2,582,475	437.24		5387.09
*(b) One-rate VAT (no cash transfers)*				
1	2,582,827	84.44		1063.42
2	2,583,828	119.02		1491.87
3	2,583,054	141.77		1779.36
4	2,582,461	161.14		2032.13
5	2,581,184	176.96		2273.48
6	2,581,761	204.02		2578.74
7	2,584,005	233.38		2947.47
8	2,581,265	260.96		3294.23
9	2,582,073	300.95		3843.83

Deciles of equivalent expenditures	Number of households	Average VAT	Average total consumption	Average cash transfer
*(c) One-rate VAT (with cash transfers)*				
1	2,582,827	− 62.88	1069.05	152.95
2	2,583,828	89.00	1499.81	37.95
3	2,583,054	151.22	1788.81	0.00
4	2,582,461	171.88	2042.87	0.00
5	2,581,184	188.76	2285.27	0.00
6	2,581,761	217.62	2592.34	0.00
7	2,584,005	248.93	2963.02	0.00
8	2,581,265	278.36	3311.63	0.00
9	2,582,073	321.02	3863.89	0.00
10	2,582,475	441.64	5385.39	0.00

Deciles of equivalent expenditures	Number of households	Average VAT	Average total consumption
*(d) Zero rate on some goods*			
1	2,582,827	69.39	1048.37
2	2,583,828	103.85	1476.70
3	2,583,054	126.54	1764.13
4	2,582,461	147.32	2018.31
5	2,581,184	165.42	2261.93
6	2,581,761	195.01	2569.73
7	2,584,005	227.03	2941.12
8	2,581,265	259.72	3293.00
9	2,582,073	306.66	3849.53
10	2,582,475	444.25	5387.99

Note: Weighted descriptive statistics. Average VAT, average total consumption, and average transfer values are in euros. In the one-rate VAT (with cash transfers) reform the average VAT is evaluated as the difference between the VAT paid and the monetary transfer received

Source: Author’s elaborations on Istat data, 2019

**Table 9:** Redistributive indices on income measure

Gini and redistributive indices	Current system	One-rate VAT (no cash transfers)	One-rate VAT (with cash transfers)	Zero rate on some goods
Gini (gross of VAT)	0.393	0.393	0.393	0.393
Gini (net of VAT)	0.396	0.398	0.394	0.396
RS index	− 0.003	− 0.005	− 0.001	− 0.003
Kakwani index	− 0.052	− 0.085	− 0.011	− 0.045
Suits index	− 0.053	− 0.089	− 0.034	− 0.047
Confidence intervals Gini gross	[0.3886 0.3975]	[0.3886 0.3975]	[0.3883 0.3978]	[0.3744 0.3903]
Confidence intervals Gini net	[0.3918 − 0.4005]	[0.3928 − 0.4036]	[0.3890 − 0.3989]	[0.3758 − 0.3966]
Confidence intervals RS (difference)	[− 0.0033 − 0.0030]	[− 0.0061 − 0.0042]	[− 0.0011 − 0.0007]	[− 0.0062 − 0.0014]

Note: Income measure is evaluated as the difference between disposable income and the VAT paid in each reform scenario. The confidence intervals are evaluated at 95% level. Source: Author’s elaborations on Istat data, 2019

**Table 10:** Concentration coefficients and redistributive indices using different take-up rates

	One-rate VAT (with cash transfers)	One-rate VAT (with cash transfers—take-up 61%)	One-rate VAT (with cash transfers—take-up 77.7%)	One-rate VAT (with cash transfers—take-up 80%)	One-rate VAT (with cash transfers—take-up 95%)
	(a)	(b)	(c)	(d)	(e)
*Concentration coefficients (ranking by equivalent expenditures net of VAT)*					
Total expenditures net of VAT	0.246	0.246	0.246	0.246	0.246
VAT	0.351	0.306	0.326	0.330	0.345
Super-reduced tax rate	–	–	–	–	–
Reduced tax rate	–	–	–	–	–
Standard tax rate	–	–	–	–	–
*Gini and redistributive indices*					
Gini (gross of VAT)	0.317	0.314	0.315	0.315	0.316
Gini (net of VAT)	0.311	0.311	0.311	0.311	0.311
RS index	0.006	0.004	0.005	0.005	0.005
Kakwani index	0.073	0.043	0.057	0.059	0.068
Suits index	0.059	0.036	0.047	0.048	0.056

Note: Column (a) reports again the results obtained with the “perfect” targeting of cash transfer. In columns (b), (c) and (d) the take-up rate used is related to the *Reddito di Cittadinanza*, while in the last column the take-up rate is related to the *Assegno Unico e Universale*. In detail: the value of 61% has been obtained from INPS data for 2020 and from the demand registered until February 2020; it is derived from Vittoria (2020). The value of 77.7% is obtained from Giuliano et al. (2022). The take-up rate is evaluated from IT-SILC data for 2017 integrated with other administrative and survey information. The value of 80% is obtained from Baldini and Gallo (2021). The take-up rate is evaluated from IT-SILC data for 2017 and integrated with other information. Finally, the value of 95% is derived from the first estimation of AUU by Biagetti et al. (2022). The data used are derived from the EU-SILC for Italy for 2019. Source: Author’s elaborations on Istat data, 2019

## Footnotes

1 A discussion of the various steps of the reform is available in Longobardi (2014).

2 See also Ebrill et al. (2001) and Mirrlees et al. (2011).

3 This point is largely recognised in the empirical literature on the impact of the zero rate in the UK. See, among other studies, the synthesis provided by de Mooji and Keen (2012), where it is also shown that a better redistributive impact could be obtained by simultaneously increasing the tax revenue from VAT.

4 See also the implicit subsidy arising from food zero-rating in Mexico, as reported in the OECD Economic Surveys of Mexico in 2007.

5 For a treatment of the issues posed by VAT in the context of the overall tax structure, see Liberati (2021).

6 This statement can be clarified by using a simple example. By considering three stages, in which a given good assumes, respectively, a value of ${\phi }_{1}$, ${\phi }_{2}$, and ${\phi }_{3}$, a VAT with a uniform tax rate $t$ will tax the first stage at ${t\phi }_{1}$, the second stage at $t\left({\phi }_{2}-{\phi }_{1}\right)$, and the third stage at $t\left({\phi }_{3}-{\phi }_{2}\right)$. Thus, the overall level of taxation will be ${t\phi }_{1}+t\left({\phi }_{2}-{\phi }_{1}\right)+t\left({\phi }_{3}-{\phi }_{2}\right)$, which is equivalent to $t{\phi }_{3}$, in turn equal to taxing the final value at the same uniform rate.

7 The C-efficiency index is defined by the ratio between actual VAT revenue (which is affected both by policy choices and by tax evasion) and the theoretical VAT revenue that could be obtained by applying the ordinary tax rate to final consumption. The index ranges from zero to one; the lower the index the less efficient VAT is compared with a uniform tax rate on final consumption.

8 See, among many studies, that of Atkinson and Stiglitz (1980), and the prior contribution by Corlett and Hague (1953).

9 On this specific topic, see Taxud (2012).

10 It is worth noting, however, that in Bulgaria the differentiation is introduced only for hotel services. In Denmark, the uniform VAT is associated with an above average exemption gap.

11 Table 5 in Appendix reports the VAT rates applied in the EU member countries and in the UK.

12 The model has been built by the authors, and is included among the tools available at CEFIP (Centre for Economic and Public Finance Research), Department of Economics, University Roma Tre. Istat does not bear any responsibility for the results presented in this paper. It should be noted that micro-data on the HBS are also available for 2020 and 2021. However, they are expected to be affected by the shock of the Covid-19 pandemic; the survey for 2019 was therefore considered preferable. Consequently, the tax legislation of that year applies as a base for the hypothetical reforms.

13 See Table 3 in Ilardi and Zanichelli (2020). See also Corte dei conti (2021).

14 Regarding the complex relationships between VAT and the issue of fraud and tax evasion, see Keen and Smith (2007).

15 For a review of the main theoretical and empirical works, see CPB (2010).

16 The list of goods considered in the analysis and the corresponding tax rates is available from the authors upon request. Some summary information on the variables used in the model is reported in Tables 6 and 7.

17 The concept of distributional characteristic can be traced back to Feldstein (1972), Ahmad and Stern (1984), and Newbery and Stern (1987).

18 The negative correlation is even weaker – as expected – when considering DC at *e* = 0.5 and at *e* = 1.

19 In Fig. 1, the points that do not belong precisely to the three VAT rates are explained by the fact that for some goods in the HBS it was necessary to calculate a weighted tax rate because of the lack of a perfect matching in the correspondence between HBS and VAT definitions.

20 The choice of the threshold at the second decile implies a certain degree of arbitrariness. Yet, when partitioning the population, the poorest 20 per cent is often used for poverty analyses, both in developed and in developing countries. Documents from the World Bank and the European Union are just some examples. In any case, the alternative of the poorest 30 per cent has also been considered, with no significant changes in the results that follow.

21 Descriptive statistics for the number of households, average amount of VAT, and average amount of total expenditures for the current system and for all hypothetical reforms are reported in Table 8.

22 Cirillo et al*.* (2021) employ the VATSIM-DF (II) micro-simulation model for their estimates. The model is implemented on a large and detailed database that includes the Household Budget Survey (HBS) and the Survey of Income and Living Conditions (SILC), supplemented by Tax Register data.

23 Equivalent expenditures are calculated by applying an equivalence scale to nominal expenditures. The scale is based on the following coefficients: 1 is assigned to the first member; 0.7 is assigned to the second member; 0.5 is assigned to all other members of the household. This scale is a simpler version of the OECD equivalence scale, which was sometimes referred to as the Oxford scale. See OECD (1982).

24 It is worth noting that in the first panel of Table 2 the concentration coefficients of monetary variables are measured over a ranking by equivalent expenditures net of VAT. We have repeated the redistributive analysis using a further measure of income. This measure is a kind of “virtual income”, and it has been evaluated as disposable income net of VAT paid in each reform scenario. The results are shown in Table 9. In line with the literature, a regressive redistributive effect emerges in all the reforms.

25 Even though the correlation between number of children and poverty is not clear-cut, we adopt the strategy that is implicitly suggested by Istat when considering poverty thresholds according to the number of household members. Thus, our cash transfer is parameterised both on the number of children and the level of expenditures below which a household can be considered poor. In any case, the situation of children in large families has often been the object of specific analyses about how to eradicate child poverty. See, for example, Bradshaw et al. (2006) for the case of UK.

26 VAT evasion due to multiple tax rates in the intermediate stages is realized, for example, by omitting the registration of sales with a higher tax rate, thus maximising the gap between credits (on purchases) and debits (on sales). See, for example, Agha and Haughton (1996) with reference to a set of efficiency problems.

27 We refer to the new poverty series releases by the Data Warehouse I.Stat.

28 See Mayshar and Yitzhaki (1996) for the basic methodology and Liberati (2001) for an application to Italian VAT.

29 The formula used for the linear decreasing cash transfer is analogous to that used in the Italian personal income tax for the number of tax credits. In our specific case, the formula is $170\left[\frac{\mathrm{1,168}-yeq}{\mathrm{1,168}}\right]$, where $yeq$ is the actual equivalent household income. Due to the non-availability of wealth data, it is not possible to implement a monetary transfer based on indicators such as ISEE (*Indicatore della Situazione Economica Equivalente*).

30 The reference is the approval of the Directive 2022/452.

31 A zero rate is extensively used in Ireland as well. Obviously, we abstract from the possible misclassification that is allowed in the presence of zero-rated goods and which may cause tax evasion. In the UK, at least in the past, a good example was provided by the practice of misclassifying adult clothing as children’s clothing, which was zero-rated. See, for example, Matthews (2003).

32 The possible impact of aggregate elasticity has also been verified in the case of the zero-rate hypothesis; in this case too, concentration coefficients are almost invariant. Results are not reported in the table.

33 For an extensive revision of the overall tax system in the direction of applying a uniform VAT see also Rossi (2017), where a tax reform with a uniform 25 per cent tax rate is applied also to income tax and other minor taxes.
